# Mendelian randomization studies of biomarkers and type 2 diabetes

**DOI:** 10.1530/EC-15-0087

**Published:** 2015-10-07

**Authors:** Ali Abbasi

**Affiliations:** 1 MRC Epidemiology Unit, University of Cambridge School of Clinical Medicine, Institute of Metabolic Science, Cambridge Biomedical Campus, Addenbrooke's Hospital, Post Box 285, Cambridge, CB2 0QQ, UK; 2 Department of Epidemiology and Department of Internal Medicine University of Groningen University Medical Center Groningen Groningen, The Netherlands

**Keywords:** biomarkers, epidemiology, Mendelian randomization, genome-wide association study, type 2 diabetes

## Abstract

Many biomarkers are associated with type 2 diabetes (T2D) risk in epidemiological observations. The aim of this study was to identify and summarize current evidence for causal effects of biomarkers on T2D. A systematic literature search in PubMed and EMBASE (until April 2015) was done to identify Mendelian randomization studies that examined potential causal effects of biomarkers on T2D. To replicate the findings of identified studies, data from two large-scale, genome-wide association studies (GWAS) were used: DIAbetes Genetics Replication And Meta-analysis (DIAGRAMv3) for T2D and the Meta-Analyses of Glucose and Insulin-related traits Consortium (MAGIC) for glycaemic traits. GWAS summary statistics were extracted for the same genetic variants (or proxy variants), which were used in the original Mendelian randomization studies. Of the 21 biomarkers (from 28 studies), ten have been reported to be causally associated with T2D in Mendelian randomization. Most biomarkers were investigated in a single cohort study or population. Of the ten biomarkers that were identified, nominally significant associations with T2D or glycaemic traits were reached for those genetic variants related to bilirubin, pro-B-type natriuretic peptide, delta-6 desaturase and dimethylglycine based on the summary data from DIAGRAMv3 or MAGIC. Several Mendelian randomization studies investigated the nature of associations of biomarkers with T2D. However, there were only a few biomarkers that may have causal effects on T2D. Further research is needed to broadly evaluate the causal effects of multiple biomarkers on T2D and glycaemic traits using data from large-scale cohorts or GWAS including many different genetic variants.

## Introduction

Over the past decade, interest in studying biological markers (biomarkers) for type 2 diabetes (T2D) has increased intensely. This happened because multiple pathobiological processes may contribute to the disease progression, which provides an opportunity to introduce preventive and therapeutic interventions for T2D (1). In clinical practice, such biomarkers (e.g., glucose and glycated haemoglobin tests) are widely used for the diagnosis of diabetes or for the monitoring of therapeutic intervention (2, 3). Targeted intervention at the biomarker level would be useful where there is evidence for a causal relationship between an exposure (like a biomarker) and T2D (4).

Traditional epidemiological studies lack sufficient information to fill the evidence gap due to unmeasured confounding or reverse causality (4, 5, 6, 7). It has been successfully shown that a complementary analysis of genetic data, termed ‘Mendelian randomization,’ has additive value to infer a causal association (4, 5, 6, 7, 8). The main assumption for Mendelian randomization is that the genetic variants do not change over time and are inherited randomly (based on Mendel's laws). In other words, the genetic variants as proxy measures for exposures (e.g., biomarkers) are essentially considered free from confounding and reverse causation. Therefore, the analysis of integrated observational-genetic data is considered similar to that of the randomized trials (9, 10). In Mendelian randomization, if there is a causal association between a biomarker and T2D, the genetic variant(s) influencing the biomarker and the outcome of interest should be associated (5, 6, 8). In the current study, evidence for causal associations between biomarkers and the risk of T2D was updated via a systematic literature search to identify Mendelian randomization studies. Next, summary data from the two largest genome-wide association studies (GWAS) for T2D or glycaemic traits (10, 11) were used to examine the effect estimates for each genetic variant compared with that of the identified studies.

## Methods

### Search strategy for candidate biomarkers

PubMed and EMBASE were searched to identify Mendelian randomization studies examining the associations between biomarkers and T2D until April 2015. The overview of this systematic literature search was conducted in accordance with the Preferred Reporting Items for Systematic Reviews and Meta-analysis (PRISMA) guidelines, when applicable (12). A manual search was also done for the references of included articles to identify other relevant studies. Because a review of published original studies was performed, the Declaration of Helsinki items related to ‘approval of medical ethics committee’ and ‘permission acquisition’ are not applicable to the current study.

### Selection criteria

Studies were included if they formally quantified a causal association between one or more biomarkers (as main exposures) and T2D (as the main outcome); used data on biomarker-associated genetic variant; and classified/defined the exposure as a biomarker that has been objectively measured in serum, plasma or urine. The following MeSH keywords or related terms were used: Diabetes mellitus, Type 2[Mesh], Type 2 Diabetes[tiab], Diabetes[ti]; and Mendelian randomization, Mendelian randomisation, instrumental variable, genetic risk score or causal association.

### Data extraction and quality assessment

**Figure 1:**
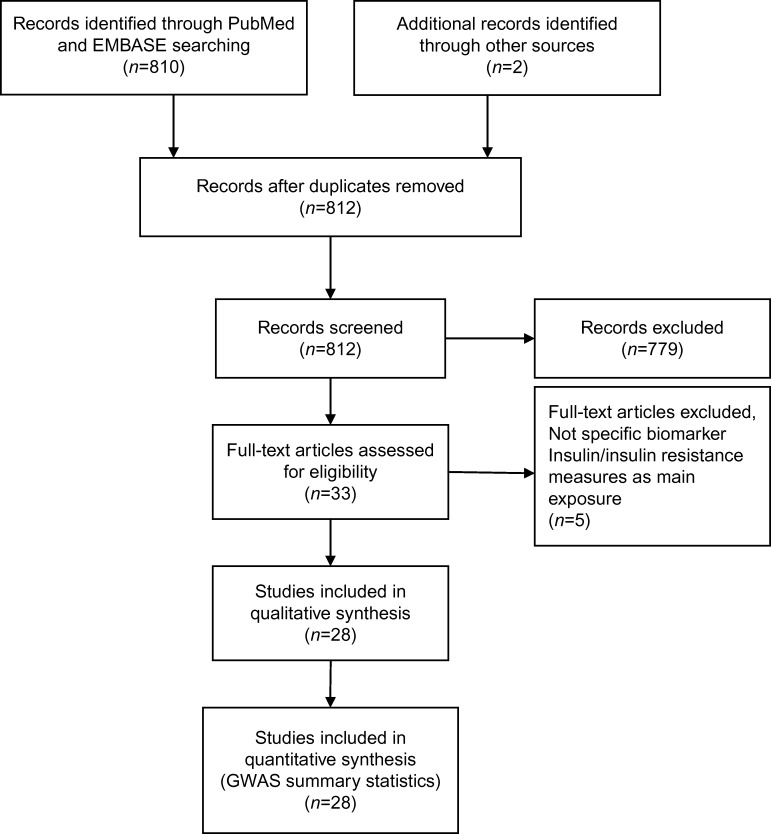
Flow diagram of the literature search.

A primary plan was made to extract necessary data from the full text of the original studies or to contact the corresponding author(s) when appropriate. Fig. 1 depicts the workflow of the literature search. T2D was determined as the main outcome if one or more of the following conditions were fulfilled: a physician diagnosed T2D as indicated by a self-report or in a primary-care database; fasting plasma glucose ≥7.0 mmol/l, a random sample plasma glucose ≥11.1 mmol/l; or the initiation of glucose-lowering medication as retrieved from a pharmacy registry or hospital records (11).

### Statistical analysis

All genetic variants that affect identified biomarkers were obtained from the original Mendelian randomization studies. To replicate the nature of the relationship between each biomarker and the outcome (i.e., T2D or glycaemic traits), a genetic approach using outcome-association data for the biomarker-related genetic variants was applied (5, 6, 8). In brief, to determine whether the same genetic variants (or a suitable proxy variant) were associated with T2D (or glycaemic traits), the corresponding summary association statistics from GWAS for T2D (DIAbetes Genetics Replication And Meta-analysis (DIAGRAMv3)) (13) and glycaemic traits (Meta-Analyses of Glucose and Insulin-related traits Consortium (MAGIC)) (14) were extracted. DIAGRAMv3 is a meta-analysis of multiple GWAS with a total number of 12 171 diabetes cases and 56 862 controls of European descent (13). Previously, details regarding the use of GWAS data for Mendelian randomization were described (5, 6, 10, 11). These selected single-test association analyses in the GWAS data are equivalent to that of individual-level data analysis (5, 6, 11).

Extracted data were tabulated on Excel spreadsheets. All statistical analyses were conducted using Excel or Stata/SE version 13.1 for Windows (http://cran.r-project.org/). A two-sided *P* value <0.05 was considered nominally significant.

## Results

### Literature search and study characteristics

After scanning 812 titles and selected abstracts, 33 articles were selected for full text review (11, 15, 16, 17, 18, 19, 20, 21, 22, 23, 24, 25, 26, 27, 28, 29, 30, 31, 32, 33, 34, 35, 36, 37, 38, 39, 40, 41, 42, 43, 44, 45, 46). The literature search process is shown in Fig. 1. Five studies were excluded as they did not measure a specific biomarker (*n*=1) or investigated a measure of insulin resistance as the main exposure (*n*=4). The characteristics of the 28 studies are summarized in Table 1.The studies were performed in different populations; most were cohort studies including middle-aged adults in the USA or Europe and were published between 2008 and 2015. Four studies were conducted only in Asian populations including Chinese or Taiwanese. In 12 studies, data from multiple GWAS were combined to examine the associations between genotypes and T2D. Six of these studies used data from DIAGRAMv2 (*n*=3) (47) or DIAGRAMv3 (*n*=3) (13), and the other six used at least two cohorts with genome-wide genotyping. DIAGRAMv2 was a meta-analysis of eight GWAS comprising 8130 individuals with T2D and 38 987 controls of European descent (47). All Mendelian randomization studies investigated only one biomarker as an exposure in relation to T2D. Some studies also examined the causal associations of a single biomarker with several other outcomes such as cardiovascular disease (18, 24), rheumatoid arthritis (18) or osteoporosis (24). In the final sample, studies included up to 28 144 T2D cases and 76 344 T2D controls or total participants.

### Biomarkers and Mendelian randomization

From 28 studies, data were retrieved on causal associations of biomarkers with T2D. In these studies, 21 unique biomarkers that were investigated at least once (16 biomarkers), twice (three biomarkers) and three times (two biomarkers) were identified (Table 1); 19 biomarkers were investigated in independent studies. However, the two biomarkers with three Mendelian randomization studies were investigated in combined studies where the same sample, e.g., DIAGRAMv2 for adiponectin (19, 43), or a part of whole cohort, e.g., European Prospective Investigation into Cancer and Nutrition (EPIC)-Potsdam for vitamin D (17, 45), were used to make total cases/controls. Eleven studies used one genetic variant as a single instrumental variable in Mendelian randomization. Eight studies used at least two independent genetic variants in the same locus as instrumental variables. The rest of the nine studies used multiple genetic variants in different loci or created a multi-locus genetic risk score for each biomarker.

In main or sensitivity analyses, four studies (11, 26, 44, 45) examined causal associations of bilirubin, lipoprotein (a), vitamin D, interleukin-1 receptor antagonist (with corresponding biomarker-associated genotypes) with T2D using the DIAGRAMv3 summary data. In Table 1, the value for each causal estimate can be interpreted as odds ratio (OR), hazard ratio (HR) or log OR (β coefficient) for diabetes per one unit change in genetically determined biomarkers or biomarker-associated genotypes. Evidence of causal association was reported for adiponectin, bilirubin, N-terminal pro B-type natriuretic peptide (NT proBNP), delta-6 desaturase (D6D), dimethylglycine, ferritin (transmembrane protease serine 6), homocysteine, macrophage migration inhibitory factor (MIF), sex hormone-binding protein (SHBG) and resistin (Table 1). For adiponectin, causal estimates were calculated as an OR of 0.86 per allele (35) or a β coefficient of 0.3 for adiponectin multi-locus genotypic risk score (19). Causal estimate for bilirubin was reported as an OR of 0.58 per 1-s.d. genetically increased log-transformed bilirubin (11). For NT proBNP (37), D6D (30), dimethylglycine (32), ferritin (22), MIF (23) and resistin (18), the expected ORs for T2D were 0.96, 0.64, 1.1, 0.79, 1.74 and 1.38 per each copy of risk alleles or genotypes, respectively. For homocysteine, causal estimates were reported as an OR of 1.29/5 μmol/l genetically increased homocysteine (24) or ORs of 1.93 and 1.52 for CC and CC/TT genotypes (25) respectively. For SHBG, the expected ORs for T2D were 0.92 per each copy of the SHBG lowering allele (34) or 0.3/1-s.d. genetically increased natural log transformed SHBG (21).

Using the GWAS data from DIAGRAMv3 or MAGIC (13, 14), associations of the genetic variants influencing these biomarkers with T2D and glycaemic traits were tested. A nominally significance (*P*<0.05) was reached for the genetic variants that affect bilirubin (*P*=0.03 for glucose and Homeostatic model assessment (HOMA)-insulin resistance (IR)), NT proBNP (*P*=0.03 for T2D), D6D activity (*P*=0.003 for T2D; *P*=2.7×10^−8^ for glucose) and dimethylglycine (*P*=0.004 for glucose) in relation to T2D or glycaemic traits (Table 2). For adiponectin, a nominally significant association with T2D or glycaemic traits was observed for seven out of 19 genetic variants. All of these seven variants, except rs12637534, were non-*ADIPOQ* adiponectin genetic variants. In line with previous Mendelian randomization studies, the overall effect of non-*ADIPOQ* variants on T2D or glycaemic traits together with null association of *ADIPOQ* variants is compatible with pleiotropic effects of adiponectin on T2D (42).

## Discussion

This literature search of Mendelian randomization studies shows here that ten out of 21 identified biomarkers were reported to be causally associated with T2D. In particular, the presence of potential causal associations (defined as nominally significant) between the biomarker-related variants and T2D and/or glycaemic traits can be confirmed for four biomarkers using the publically available GWAS data. The inconsistency between the identified studies and the summary data from DIAGRAMv3 or MAGIC can be to some extent explained by different design and populations applying across studies, the possibility of false positive associations, selection bias, the heterogeneity in T2D, the use of varied sources to ascertain T2D cases, differences in data quality control and pre-analysis preparations (6, 11).

Taken together, these findings support that the oxidative stress system (bilirubin or metabolites of bilirubin), the brain natriuretic peptide (BNP) hormone system, D6D activity and dimethylglycine may contribute to the development of diabetes or insulin resistance through secondary effects (i.e., pleiotropy) or direct mechanisms (11, 37, 42). Bilirubin that is the major end-product of heme catabolism has antioxidant properties and may compensate the oxidative stress (11, 48). Oxidative stress has been shown as an important factor in the pathophysiology of diabetes (11). At the cellular level, bilirubin can be oxidized to its precursor, biliverdin, to detoxify the excess of oxidants. Biliverdin is rapidly recycled to bilirubin via the action of biliverdin reductase, generating a physiologic cytoprotective cycle in several tissues (49). The underlying mechanism of a protective role of BNP in the aetiology of T2D is unknown in humans (37). In mice, overexpressed BNP signalling cascade can protect against diet-induced insulin resistance and obesity through promoting muscle mitochondrial biogenesis and fat oxidation (37, 50). The biological mechanisms of the relationship between D6D activity and T2D are not well understood (30). Although data of human experimental studies are scarce, observational studies have shown that D6D activity or lifestyle-induced changes in D6D activity was associated with insulin resistance (30, 51, 52). Because D6D catalyses the synthesis of fatty acids, one can speculate that the link between D6D activity and T2D is likely to be mediated by changes in fatty acid composition, which in turn may affect insulin signalling and receptor-binding affinities (30). Dimethylglycine is metabolized to glycine by dimethylglycine dehydrogenase (DMGDH) in mammals (32). Accordingly, a recent GWAS identified that the *DMGDH* genetic variants were strongly associated with blood-based dimethylglycine (53). Epidemiological studies have observed an inverse association between the precursor of dimethylglycine, betaine, and metabolic risk factors (54) but a positive association of elevated glycine with increased insulin sensitivity (32). In humans, cardiometabolic effects of the inhibition of DMHDH or the supplementation of dimethylglycine have not been investigated (32). However, experimental animal studies have suggested a protective role of dimethylglycine in glucose metabolism through a reduction in DMGDH function (32, 55, 56).

In the post-omics era, epidemiological studies basically suggest that the levels of a given biomarker differ between patients with T2D (or the individuals at high risk for developing diabetes) and individuals without diabetes (2, 57). If the biomarker is not causally related to the disease outcome, the process of developing diabetes may cause the increase or decrease in the levels of the biomarker, as one of the T2D consequences, called reverse causality (4, 26, 37). Unmeasured confounding or measured confounding factors with errors (for example, physical activity by self-report) is another explanation for the observed associations between most biomarkers and T2D. In this context, the use of genetic data (as in Mendelian randomization) can enhance the likelihood that the association between a biomarker and T2D is causal or not (4, 36, 37). The potential role of biomarkers in the development of T2D and the trajectories of glycaemic traits using longitudinal analysis remains to be further confirmed (6, 11). The latter analytical approach can provide insight into the potential value of biomarkers, which indicates pathobiological signals of metabolic changes in the aetiology of T2D. T2D is influenced by the interactions of multiple genes or a gene may have been mapped to multiple biomarkers rather than the biomarker of interest (4, 13, 14). Thus, an extensive knowledge of gene function and biological processes and that the genome interacts with environmental factors is needed to better understand how genetic variations in the human genome contributes to lifelong risk of T2D (4, 11, 13, 14, 57).

In this review, most studies only investigated a single biomarker-diabetes association, and statistically significant associations are reported more often. Here, publication bias should be considered. Other limitations include the lack of complementary analyses in the genetic associations for glycaemic traits and that set of genetic variants or large-scale GWAS for biomarkers were scarce. Similarly, the Mendelian randomization approach using GWAS summary statistics can be extended as a secondary analysis of several datasets that have data on the biomarker-associated variants (or their proxies) for several diseases or traits. This multidisciplinary research requires that a large group of consortia are contacted to obtain summary association statistics from GWAS consortia for outcomes of interest. Moreover, for the biomarkers linked to T2D, underlying biological mechanisms remain unknown. To uncover the underlying mechanisms, one needs to perform a complementary strand of experimental research. Before that, an *in silico* functional gene network (pathway) analysis can be used to speculate on the possible biological mechanisms of biomarker-disease associations (58, 59). Finally, Mendelian randomization cannot completely control for the possibility of developmental compensation, called canalization, confounding and pleiotropic effects (5, 6, 8, 11, 37).

In conclusion, this is the first study that updates evidence for causal associations between biomarkers and T2D to date. Several Mendelian randomization studies investigated the nature of associations of biomarkers with T2D. Most biomarkers were investigated in a single cohort study or population. However, there were only a few biomarkers that may have causal effects on T2D. Further research is warranted to broadly evaluate the causal effects of multiple biomarkers on T2D and glycaemic traits using data from large-scale cohorts or combined GWAS including many different genetic variants. This genetic approach may advance our understanding of the causes of T2D and potentially enable us to explore novel targets for the prevention and treatment of diabetes.

## Figures and Tables

**Table 1:** Mendelian randomization studies for biomarkers and type 2 diabetes.

**Biomarkers**	**No. of study**	**Study**	**Country/year**	**Cases/total or controls**	**Genetic variant(s)/locus**	**Causal estimate**	**Type of associations**
Adiponectin	3 (19, 35, 42)	DIAGRAMv2, ARIC, FUSION, WTCCC, EPIC-InterAct	US, Europe/2013	15 960/64 731	rs17300539, rs1736653, rs3774261, rs3821799/*ADIPOQ*	Null	
		BHS, CUDAS, FDS	Australia/2013	967/2355	rs12637534, rs16861209, rs17366568/*ADIPOQ*	OR, 0.86	Per allele
		DIAGRAMv2	US, Europe/2012	–/22 044	rs3001032/*LYPLAL1*, rs1108842/*GNL3*, rs1597466/*TSC22D2*, rs6810075/*ADIPOQ*, rs998584/*VEGFA*, rs2980879/*TRIB1*, rs7955516/*PDE3A*, rs601339/*GPR109A*, rs7133378/*DNAH10*, rs2925979/*CMIP*, rs12922394/*CDH13*, rs731839/*PEPD*	β, −0.30	Average additive effect of adiponectin- raising alleles
Beta-carotene	1 (33)	WTCCC, DGI, FUSION	USA, Europe/2009	4549/5579	rs6564851/*BCMO1*	Null	
Bilirubin	1 (11)	PREVEND	Netherlands/2015	210/3381	rs6742078/*UGT1A1*	OR, 0.58	1-s.d.
N-terminal pro B-type natriuretic peptide	1 (37)	DIAGRAMv2, Norfolk Diabetes, Cambridgeshire, ADDITION-Ely, French, Swiss 1, Swiss 2, Austrian, MONICA, INCA, UK	US, Europe/2011	23 382/57 898	rs198389/*NPPB*	OR, 0.96	*C* allele
C-reactive protein	1 (16)	Whitehall II	UK/2008	354/5274	rs1130864, rs1205, rs3093077/ *CRP*	Null	
Delta-6 desaturase	1 (30)	EPIC-Potsdam	Germany/2011	673/2724	rs174546/*FADS1*	RR, 0.64	*TT* genotype
Dimethylglycine	1 (32)	MDC-CC, MDC, MPP	Sweden/2015	4201/33 898	rs2431332/ *DMGDH*	OR, 1.1	*A* allele
Ferritin/TMPRSS6	1 (22)	Beijing	China/2012	272/1574	rs855791, rs4820268/ *TMPRSS6*	OR, 0.79	*AG* haplotype
Fetuin-a	1 (27)	CHS	US/2013	259/3093	rs4917, rs2248690/*AHSG*	Null	
Homocysteine	2 (24, 25)	Hangzhou	China/2014	774/500	rs1801131/*MTHFR*, rs4646356/*PEMT*	OR, 1.93, 1.52	*CC*, *CT*+*TT*
		–	Europe, Asia, Africa/2013	4011/4303	rs12134663, rs1801133/*MTHFR*	OR, 1.29	5 μmol/l
IL-1Ra	1 (26)	DIAGRAMv3 and EPIC-InterAct	US, Europe/2015	18 715/61 692	rs6743376, rs1542176/*IL1RN*	Null	
Leukocyte telomere length	1 (46)	WHI-OS	US/2012	1675/2382	rs34368910/*TRF1*, rs4888444/*TRF2*, rs4975605/*TERT*, rs938886, rs2228041,rs12880583/*TPP1*	Null	
Lipoprotein(a)	2 (29, 44)	Danish general population	Denmark/2014	2157/28 567	rs10455872/*LPA*	Null	
		DIAGRAMv3,EPIC-Norfolk	USA, Europe/2014	10 088/68 346	rs10455872/*LPA*	Null	
Macrophage migration inhibitory factor	1 (23)	MONICA/KORA Augsburg	Germany/2008	502/1632	rs1007888/*MIF*	HR, 1.74	*C* allele
miRNAs	1 (41)	Harbin	China/2015	995/967	rs895819/miR-27a, rs531564/miR-124a, rs2910164/miR-146a	Null	
Resistin	1 (18)	CVDFACTS	Taiwan/2014 US, Europe/2010	230/3400	rs3745367,rs1423096/*RETN*	OR, 1.38	*GG*
Sex hormone-binding globulin	2 (21, 34)	WTCCC, Dundee, EFS-Y2TD, Danish, DGI, MCC, FUSION 1-2, KORA, Cambridgeshire, ADDITION-Ely, NDCCS, DeCODE, METSIM, DIAGEN		27 657/58 481	rs1799941/*SHBG*	OR, 0.92	*G* allele
		WHS	USA/2009	359/359	rs6259/*SHBG*	OR, 0.3	1-s.d.
Triglycerides	1 (20)	Go-DARTS	UK/2011	5637/6860	rs2954029/ *TRIB1*, rs714052/*MLXIPL*, rs7557067/*APOB*, rs17216525/*NCAN*, rs10889353/*ANGPTL3*, rs7679/*PLTP*, rs7819412/*XKR6-AMAC1L2*, rs328/*LPL*, rs3135506/*APOA5*, rs662799/*APOA5*	Null	
Uric acid	1 (36)	Cambridgeshire, ADDITION-Ely, Norfolk Diabetes	UK/2011	7504/8560	rs12129861/*PDZK1*, rs742132/*LRRC16A*, rs505802/*SLC22A12*, rs12356193/*SLC16A9*, rs17300741/*SLC22A11*, rs1165151/*SLC17A1*, rs2231142/*ABCG2*, rs734553/ *SLC2A9*	Null	
Vitamin D	3 (17, 28, 45)	DIAGRAMv3, ADDITION-Ely, Norfolk Diabetes, Cambridgeshire, EPIC-InterAct	US, Europe/2015	281 44/76 344	rs12785878/DHCR7, rs10741657/CYP2R1, rs4588/DBP, rs17217119/CYP24A1	Null	
		EPIC-Potsdam	Germany/2013	1572/2121	rs2282679, rs1155563/GC, rs12785878, rs3829251/DHCR7, rs10741657/CYP2R1, rs6013897/CYP24A1, rs6599638/Ch10orf88, rs10877012/CYP27B1	Null	
		Tromsø	Norway/2012	1092/9528	rs2298850/*DBP GC*, rs10741657/CYP2R1, rs3794060/NADSYN1, rs6013897/*CYP24A1*	Null	
Vitamin D binding protein	1 (31)	CaMos	Canada/2014	201/2254	rs2282679/*DBP GC*	Null	

RR, relative risk; IL1-Ra, interleukin 1 receptor antagonist; DIAGRAM, DIAbetes Genetics Replication And Meta-analysis; EPIC-InterAct, European Prospective Investigation into Cancer and Nutrition (EPIC)-InterAct; ARIC, Atherosclerosis Risk In Communities; WTCCC, Welcome Trust Case Control Cohort; FUSION, Finland-US Investigation of NIDDM genetics; BHS, Busselton Population Health Survey; CUDAS, Carotid Ultrasound Disease Assessment Study; FDS, Fremantle Diabetes Study; PREVEND, Prevention of Renal and Vascular ENd-stage Disease; iNCA, iNsuffisance CAdiaque; MONICA, Multinational MONItoring of trends and determinants in CArdiovascular disease; MDC, Malmö Diet and Cancer Study; MDC-CC, Malmö Diet and Cancer Cardiovascular Cohort; MPP, Malmö Preventive Project; CVDFACTS, CardioVascular Disease risk FACtors Two-township Study; KORA, Kooperative Gesundheitsforschung in der Region Augsburg; EFS-YT2D, Exeter Family Study-Young-onset T2D; NDCCS, Norfolk Diabetes Case Control Study; METSIM, METabolic Syndrome In Men; DIAGEN, DIAbetes GENetic study; CHS, Cardiovascular Health Study; WHI-OS, Women's Health Initiative Observational Study Cohort; ADDITION, Anglo-Danish-Dutch Study of Intensive Treatment In People with Screen Detected Diabetes in Primary Care; Go-DARTS, Genetics of Diabetes Audit and Research in Tayside Scotland; CaMos, Canadian Multicentre Osteoporosis Study.

**Table 2:** Nominally significance for each biomarker-associated genetic variants with glucose, HOMA-IR and the risk of type 2 diabetes.

Biomarkers	Genetic variants	*P* value*			Nominally significance
T2DM^13^	Glucose^14^	HOMA-IR^14^
Adiponectin	rs1736653	–	–	–	NA
	rs2980879	0.96	0.96	0.93	Null
	rs16861209	0.89	0.85	0.51	Null
	rs601339	0.89	0.83	0.03	Yes
	rs17300539	0.87	0.81	0.15	Null
	rs7955516	0.86	0.84	0.83	Null
	rs12922394	0.54	0.07	0.19	Null
	rs3821799	0.51	0.19	0.80	Null
	rs731839	0.41	0.34	0.01	Yes
	rs3774261	0.32	0.44	0.76	Null
	rs17366568	0.31	0.97	0.40	Null
	rs7133378	0.16	0.21	0.33	Null
	rs12637534	0.15	0.38	0.00	Yes
	rs6810075	0.1	0.77	0.52	Null
	rs998584	0.06	0.81	0.68	Null
	rs1108842	0.032	0.47	0.05	Yes
	rs3001032	0.029	0.27	0.00	Yes
	rs1597466	0.009	0.06	0.47	Yes
	rs2925979	0.002	0.05	0.18	Yes
Beta-carotene	rs6564851	0.22	0.39	0.27	Null
Bilirubin	rs6742078	0.1	0.02	0.03	Yes
NT proBNP	rs198389	0.03	0.37	0.39	Yes
CRP	rs1205	0.66	0.68	0.64	Null
	rs3093077	0.048	0.95	0.39	Yes
	rs1130864	–	0.12	0.77	Null
D6-desaturase	rs174546	0.003	2.7×10^−8^	0.89	Yes
Dimethylglycine	rs2431332	0.51	0.004	0.29	Yes
Ferritin/TMPRSS6	rs855791	1	0.09	0.25	Null
	rs4820268	0.73	0.15	0.13	Null
Fetuin-a	rs4917	0.08	0.11	0.67	Null
	rs2248690	0.055	0.96	0.77	Null
Homocysteine	rs4646356	0.88	0.43	0.35	Null
	rs12134663	0.78	0.69	0.62	Null
	rs1801131	0.22	0.61	0.24	Null
	rs1801133	0.10	0.46	0.43	Null
IL-1Ra	rs1542176	0.64	0.008	0.71	Yes
	rs6743376	–	0.91	0.46	Null
LTL	rs12880583	–	–	–	NA
	rs2228041	–	–	–	NA
	rs34368910	–	–	–	NA
	rs938886	0.88	0.042	0.74	Yes
	rs4975605	0.66	0.45	0.61	Null
	rs4888444	0.09	0.91	0.34	Null
Lp(a)	rs10455872	0.36	0.42	0.32	Null
MIF	rs1007888	0.39	0.22	0.94	Null
miRNAs	rs12610873	0.033	0.80	0.71	Yes
	rs531564	0.66	0.53	0.97	Null
	rs2910164	0.52	0.01	0.36	Yes
Resistin	rs3745367	0.77	0.99	0.58	Null
	rs1423096	0.72	0.81	0.34	Null
SHBG	rs12150660	0.18	0.52	0.24	Null
	rs6259	0.94	0.42	0.35	Null
Triglycerides	rs12285095	0.41	0.97	0.62	Null
	rs7557067	0.9	0.78	0.15	Null
	rs7679	0.88	0.90	0.29	Null
	rs2954029	0.72	0.64	0.87	Null
	rs662799	0.28	0.04	0.55	Yes
	rs714052	0.28	0.81	0.80	Null
	rs10889353	0.19	0.42	0.21	Null
	rs7819412	0.08	0.80	0.01	Yes
	rs328	0.03	0.05	0.04	Yes
	rs17216525	0.00	0.98	0.81	Yes
Uric acid	rs1165151	0.74	0.42	0.86	Null
	rs2231142	0.73	0.83	0.36	Null
	rs505802	0.62	0.60	0.62	Null
	rs734553	0.57	0.20	0.72	Null
	rs742132	0.25	0.50	0.91	Null
	rs12356193	0.14	0.95	0.47	Null
	rs12129861	0.08	0.94	0.50	Null
	rs17300741	0.03	0.34	0.36	Yes
Vitamin-D	rs10877012	–	–	–	NA
	rs3755967	0.74	0.85	0.19	Null
	rs1155563	0.93	0.77	0.06	Null
	rs2298850	0.66	0.89	0.15	Null
	rs3829251	0.54	0.09	0.26	Null
	rs3794060	0.34	0.28	0.10	Null
	rs6599638	0.30	0.17	0.43	Null
	rs10741657	0.23	0.37	0.08	Null
	rs12785878	0.14	0.32	0.17	Null
	rs17217119	0.06	0.36	0.21	Null
	rs6013897	0.06	0.33	0.12	Null
Vitamin D-BP	rs2282679	0.76	1.00	0.12	Null

*P values for each of the biomarker variants were extracted from publicly available meta-analyses of genome-wide association studies (13, 14). NA, not applicable; CRP, C-reactive protein; BNP, brain natriuretic peptide; interleukin 1 receptor antagonist (IL1-Ra); Lp(a), Lipoprotein(a); LTL, leukocyte telomere length; MIF, macrophage migration inhibitory factor; NT proBNP, N-terminal pro B-type natriuretic peptide; SHBG, sex hormone binding globulin, Vitamin D-BP, Vitamin D binding protein.
